# Synthesis, Crystal Structure, and Biological Evaluation of a Series of Phloretin Derivatives

**DOI:** 10.3390/molecules191016447

**Published:** 2014-10-13

**Authors:** Li Wang, Zheng-Wei Li, Wei Zhang, Rui Xu, Fei Gao, Yang-Feng Liu, Ya-Jun Li

**Affiliations:** 1 Department of Scientific Research, The Affiliated Hospital of Xi’an Medical University, Xi’an 710077, China; 2 Department of Anesthesia, The Affiliated Hospital of Xi’an Medical University, Xi’an 710077, China; 3 Department of Neurology, The Affiliated Hospital of Xi’an Medical University, Xi’an 710077, China; 4 Department of Neurology, The People’s Liberation Army No. 451 Hospital, Xi’an 710032, China

**Keywords:** phloretin derivatives, X-ray diffraction analysis, mechanism, anticancer activity

## Abstract

A one-step synthesis of phloretin derivatives **2**–**11** from phloretin in good to excellent yields is reported. Their structures were characterized by ^1^H-NMR, ^13^C-NMR and MS, and the structures of **8** and **11** were determined by X-ray diffraction analysis. A mechanism for the formation of **9**–**11** is proposed. Compared with the anticancer drug docetaxel, phloretin, phloretin derivatives and phlorizin exhibited moderate cytotoxicity toward the MDA-MB-231, SPC-A1, A549, MCF-7 and EC109 cell lines. Among all of the tested compounds, **7** exhibited the strongest cytotoxicity toward the five cell lines and was more active than docetaxel in MDA-MB-231 cells. Our findings suggest that these derivatives hold great promise for further development as anticancer agents.

## 1. Introduction

**Figure 1:**
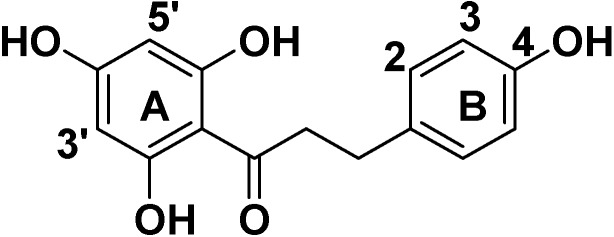
The structure of phloretin.

**Figure 2:**
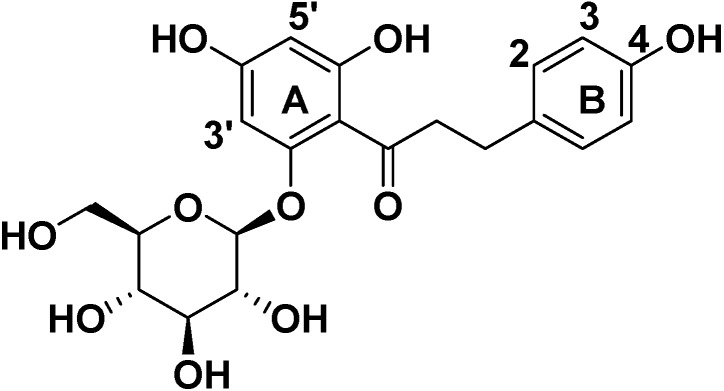
The structure of phlorizin.

Phloretin, a naturally occurring flavonoid, belongs to the dihydrochalcone chemical class and consists of a C6-C3-C6 skeleton structure (two aromatic rings connected by a C3 chain, Figure 1) [1,2,3]. Phloretin is primarily found in apples, pears, and other succulent fruits. It exhibits various biological and pharmacological activities, such as anti-inflammatory, anticancer [4], antimutagenic and anticarcinogenic activities, and has been used as a cosmeceutical ingredient [5,6,7]. Recently, its antitumor properties have also attracted much attention from pharmacologists. Many studies indicate that phloretin inhibits the growth of human leukemia, bladder cancer, and rat mammary adenocarcinoma cells *in vivo* and induces apoptosis of B16 murine melanoma and HL60 human leukemia cells [8,9]. Phloretin also exerts a strong inhibitory effect on the proliferation of SMMC-7721, HT-29 and human liver cancer cell lines [10,11,12]. Phloretin can be used for the treatment of hepatomas and other tumors; it is therefore currently being evaluated as a potential chemotherapeutic agent. Phlorizin (phloretin-2'-*O*-glucoside, Figure 2) is the product of phloretin glycosylation. Since its discovery more than 170 years ago, phlorizin has been widely investigated in biofilm research and the field of human medicine [13,14,15,16,17,18].

Phloretin and its derivatives have a broad and important role in various fields of human health and have become a focus of natural products research. Although numerous studies show that phloretin has anticancer effects, its solubility in lipids and water is poor, the interaction target of phloretin is unclear, and its pharmacodynamic effects are not optimal. In addition, little research has been performed regarding the antitumor activities of phloretin derivatives, except for phlorizin and its derivatives. Therefore, to improve the bioavailability of phloretin and investigate the antitumor activities of phloretin derivatives compared with docetaxel, the synthesis of a series of phloretin derivatives was accomplished and their antitumor activity has been evaluated against various cancer cell lines, including A549 (human lung cancer cell line), SPC-A1 (human non-small-cell lung cancer cell line), EC109 (human esophageal cancer cell line), MCF-7 (human breast adenocarcinoma cell line) and MDA-MB-231 (human breast cancer cell line) cells.

## 2. Results and Discussion

**Scheme 1:**
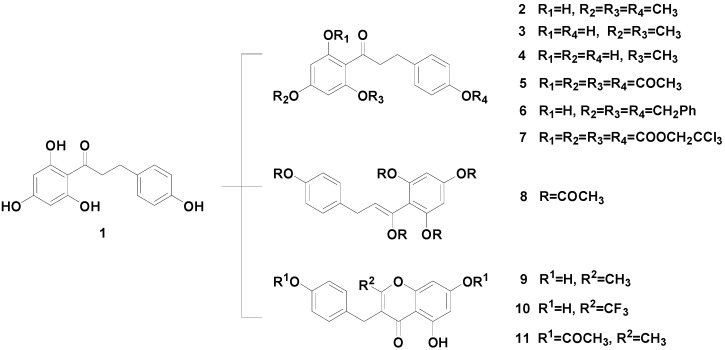
Synthesis of phloretin derivatives **2**–**11**.

**Figure 3:**
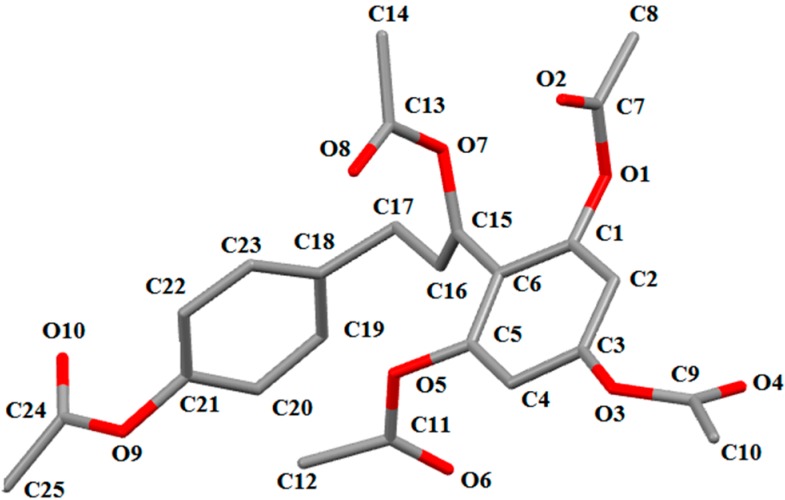
The molecular structure of **8**, showing the atom numbering scheme. For the sake of clarity, H atoms have been omitted. Displacement ellipsoids are drawn at the 30% probability level.

**Figure 4:**
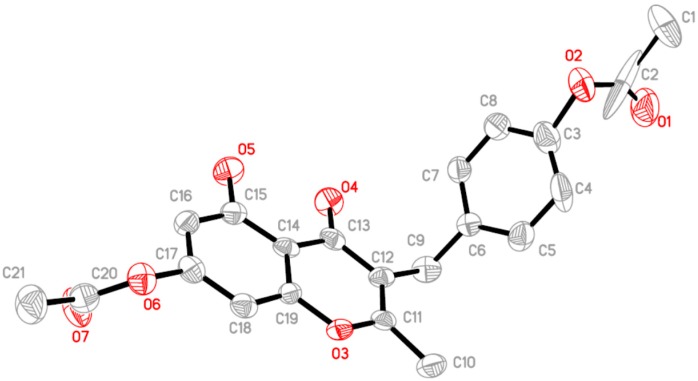
The molecular structure of **11**, showing the atom numbering scheme. For the sake of clarity, H atoms have been omitted. Displacement ellipsoids are drawn at the 30% probability level.

The synthesis of a series of phloretin derivatives is outlined in Scheme 1, starting from phloretin (**1**), which is commercially available. By varying the reaction temperature, **2**–**4** were synthesized by methylation of the phenolic hydroxyl groups of phloretin. A mixture of phloretin (**1**, 10 mmol) and acetic anhydride (50 mmol) was refluxed for 5 h to give **5** in 85% yield. Under different reaction conditions, a mixture of phloretin **1** (10 mmol) and acetic anhydride (50 mmol) was stirred under reflux overnight in the presence of triethylamine (50 mmol). The progress of the reaction was monitored by thin-layer chromatography (TLC). During the reaction, three new products whose structures differed completely from that of **5** were detected by TLC. The three products (compounds **8**, **9** and **11**) were isolated in 19%, 5.1% and 66% yields, respectively. The carbonyl of phloretin had isomerized to the enol form, which was acetylated to afford **8**. Compound **10** was synthesized using a similar method. Phloretin was treated with PhCH_2_Br to give **6**. Compound **7** was readily prepared by acylation of phloretin with 2,2,2-trichloroethyl chloroformate. The new compounds **7** and **10** were characterized by MS, ^1^H-NMR and ^13^C-NMR. Compounds **8** and **11** were also characterized by X-ray diffraction analysis; their structures are shown in Figure 3 and Figure 4.

*Reagents and conditions*: **2**: (CH_3_)_2_SO_4_, K_2_CO_3,_ (CH_3_)_2_CO, r.t., 3 h, 80%; **3** and **4**: (CH_3_)_2_SO_4_, K_2_CO_3,_ (CH_3_)_2_CO, 0 °C, 3 h, 65% for **3** and 21% for **4**; **5**: (CH_3_CO)_2_O, reflux, 5 h, 85%; **6**: PhCH_2_Br, K_2_CO_3_, (CH_3_)_2_CO, 0 °C to r.t., 5 h, 50%; **7**: Cl_3_CCH_2_OCOCl, K_2_CO_3_, (CH_3_)_2_CO, 0 °C to r.t., 80%. **8**, **9** and **11**: (CH_3_CO)_2_O, (CH_3_CH_2_)_3_N, reflux, overnight, 19% for **8**, 5.1% for **9** and 66% for **11**. **10**: (CF_3_CO)_2_O, (CH_3_CH_2_)_3_N, reflux, overnight, 78%.

The ^1^H-NMR spectra of **2**–**4** show chemical shifts at 14.028, 14.094 and 12.321 ppm, respectively; these shifts are assigned to the 2-OH and are farther downfield than the usual value of 9.91 ppm because of intramolecular hydrogen bonding. The same explanation is applicable to the chemical shifts of 14.037, 12.950, 12.200 and 12.852 ppm observed in **9**–**11**, respectively.

In the crystal structure of **8**, the dihedral angle between the two phenyl rings is 87.91°. In the intermolecular interaction C25-H25A…O3, the H25A…O3 distance is 2.694 Å, which is slightly shorter than the sum of the van der Waals radii of 2.72 Å, whereas the C20-H20…O2 distance of 2.499 Å is shorter than the C25-H25A…O3 distance. These results suggest that the C20-H20…O2 interaction is quite strong and is the greatest contributor to the stability of the molecules.

In a stereoview of the crystal structure of **11**, several weak C-H...O interactions of the types C_aryl_-H…O and C_sp_^3^-H…O are present, which causes the molecule to dimerize. The molecules are linked by weak C_aryl_-H…O hydrogen bonds, and C18-H18 and C25-H25 interact with the carbonyl O atoms (O4 and O11, respectively) of different neighboring molecules. The new formed ring (O3\C11\C12\C13\C14\C19) is nearly planar, with a dihedral angle of 2.30°. The phenyl rings C14-C19 (centroid *Cg*1) and C24-C29 (centroid *Cg*2) are involved in π–π interactions, with a *Cg*1…*Cg*2 centroid-centroid distance of 3.660 Å, which makes them nearly coplanar, with a dihedral angle of 4.77°. These hydrogen bonds and other interactions appear to play an important role in controlling the molecular conformation.

**Scheme 2:**
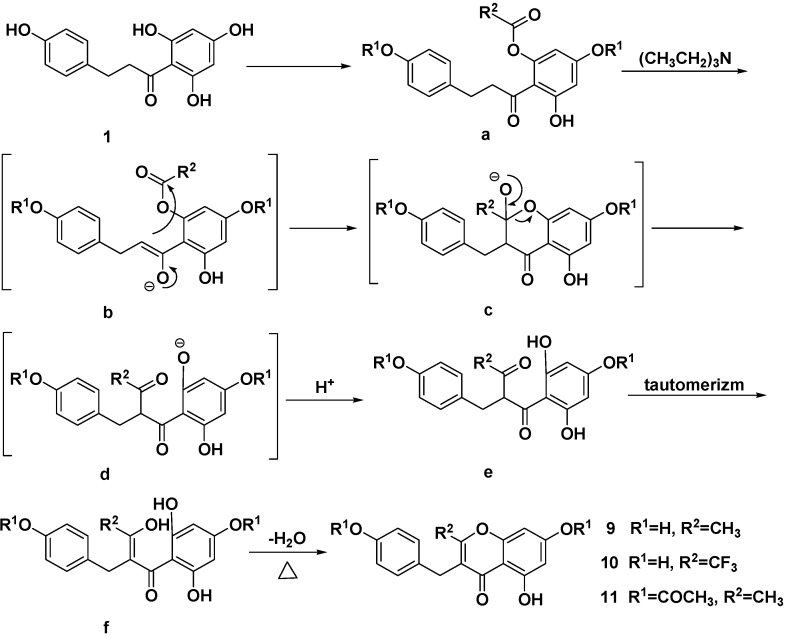
A proposed mechanism of formation for **9**–**11**.

Much attention has been devoted to the structures of **9**–**11**. A proposed mechanism of their formation, from a theoretical perspective, is presented in Scheme 2. Phloretin (**1**) can react with the acid anhydride to give intermediate **a**, in which the α-hydrogen of the carbonyl group can be deprotonated in the presence of triethylamine (acting as a base) to form enolate **b**. The α-carbon of the negatively charged enolate then attacks the carbonyl carbon of the nearby acetyl group through an addition-elimination reaction to afford intermediate **d**. Intermediate **d** is protonated to give **e**. Intermediate **e**, as a 1,3-dione, readily interconverts to the enol form **f**. Finally, **9**–**11** are formed by an intramolecular dehydration reaction of intermediate **f** under heating.

Phloretin and its derivatives were screened *in vitro* for their antitumor activities against A549, SPC-A1, EC109, MCF-7 and MDA-MB-231 cells using the standard MTT method, with the antitumor drug docetaxel used as a positive control. The *in vitro* cytotoxicity screening assays were conducted at different compound concentrations. All of the experiments were carried out in triplicate. The IC_50_ values were calculated from the percentage of cytotoxicity by nonlinear curve fitting and are presented in Table 1.

Among all of the investigated compounds, **7** exhibits the most potent growth inhibition of MDA-MB-231 cells, with an IC_50_ value of 0.839 μM, indicating that it is more potent than docetaxel, which exhibited an IC_50_ value of 0.998 μM. Except for **7**, all of the compounds exhibit systematic cytotoxicity in MDA-MB-231 cells, and their cytotoxicities are weaker than that of docetaxel. Moreover, the cytotoxicities of phloretin and phlorizin are lower than that of docetaxel in MDA-MB-231 cells. Approximately 40% of the compounds inhibit cell growth in A549 cells, and the cytotoxicities of **3** (IC_50_ = 19 μM) and **4** (IC_50_ = 23 μM) are 3.7- and 3-fold greater than that of docetaxel (IC_50_ = 70 μM), respectively. The cytotoxicity of **7** (IC_50_ = 51 μM) is stronger than that of docetaxel, whereas the cytotoxicities of phloretin and phlorizin are weaker than that of docetaxel.

Compared with docetaxel, all of the compounds exhibit better growth inhibition of SPC-A1 cells. For example, the anti-proliferative effects of **3** (IC_50_ = 33 μM) and **7** (IC_50_ = 47 μM) are 5- and 3.2-fold, respectively, greater than that of docetaxel (IC_50_ = 149 μM), and the cytotoxicities of phloretin and phlorizin are similar to that of docetaxel. With respect to EC109 cells, **3**, **4** and **7** exhibit better cytotoxicities than docetaxel. The cytotoxicities of the remaining compounds are generally weaker than that of docetaxel. In contrast, most of the compounds exhibit are relatively active toward MCF-7 cells, but are less active than docetaxel. With an IC_50_ value of 10 μM, **7** exhibits significantly weaker activity compared to docetaxel (IC_50_ = 1.46 μM) in MCF-7 cells.

**Table 1:** *In vitro* anti-proliferative activity of **1**–**11** against human cancer cell lines.

Compound	IC_50_ (μM)				
	MDA-MB-231	A549	SPC-A1	EC109	MCF-7
**1**	30.54 ± 1.95	133 ˃ 100	147 ˃ 100	148 ˃ 100	78.64 ± 0.86
**2**	14.73 ± 1.28	132 ˃ 100	131 ˃ 100	138 ˃ 100	117 ˃ 100
**3**	14.43 ± 0.99	18.79 ± 0.54	32.90 ± 1.13	30.35 ± 0.76	8.42 ± 0.74
**4**	6.93 ± 0.78	23.15 ± 0.92	39.29 ± 0.78	31.79 ± 1.09	137 ˃ 100
**5**	40.886 ± 1.94	103 ˃ 100	94.35 ± 1.25	109 ˃ 100	104 ˃ 100
**6**	37.615 ± 1.15	105 ˃ 100	96.40 ± 0.38	82.48 ± 1.18	66.21 ± 1.22
**7**	0.839 ± 0.83	50.81 ± 0.66	47.25 ± 0.93	42.14 ± 0.88	10.16 ± 1.04
**8**	33.593 ± 0.67	95.43 ± 0.83	101 ˃ 100	95.41 ± 1.39	12.49 ± 0.89
**9**	31.69 ± 1.64	62.80 ± 0.96	91.5 ± 1.63	87.26 ± 0.91	88.73 ± 1.62
**10**	21.27 ± 1.36	113 ˃ 100	94.50 ± 1.36	103 ˃ 100	111 ˃ 100
**11**	92.168 ± 0.86	110 ˃ 100	97.20 ± 1.45	107 ˃ 100	108 ˃ 100
**Phlorizin**	3.649 ± 1.19	120 ˃ 100	133 ˃ 100	116 ˃ 100	15.99 ± 0.67
**Docetaxel**	0.998 ± 1.04	70.30 ± 1.06	149 ˃ 100	56.44 ± 1.62	1.46 ± 1.52

In summary, **7** exhibits the most potent growth inhibition in most cell lines, except for MCF-7, compared to docetaxel. Interestingly, **7** contains 12 chlorine atoms, which suggests that chloro substituents may play a role in increasing its cytotoxicity. Holla *et al.* synthesized a series of 2-chloro-1,4-bis-(5-substituted-1,3,4-oxadiazol-2-ylmethyleneoxy)phenylene derivatives and evaluated their anticancer activities [19]. Their research showed that compounds that contain two or more chloro substituents exhibit better anticancer activities than compounds with less than two chloro substituents, which is a result similar to ours. Furthermore, our results also show that **3**–**8** exhibit better anti-proliferative effects than phloretin in the five cell lines tested. The molecular structure of phloretin includes four hydroxyl groups. With an increase in hydroxyl groups, the cytotoxicity increases. We conclude that **7** exhibits broad-spectrum antitumor activity worthy of further pharmacological investigation.

The growth inhibitory effects of all compounds on MDA-MB 231, SPC-A1, A549, MCF-7 and EC109 cell lines were measured by the MTT assay after 3 days of incubation. The IC_50_ values, defined as the drug concentration at which 50% of the cells are viable, were calculated from the respective logarithmic cytotoxicity curves of the different cancer cells. The data are shown as the means ± SD from three independent experiments.

## 3. Experimental Section

### 3.1. General Information

The ^1^H-NMR and ^13^C-NMR spectra were recorded on a Bruker 300 MHz Fourier-transform (FT)-NMR spectrometer, and chemical shift values are reported as δ ppm relative to tetramethylsilane (TMS) in CDCl_3_ or DMSO-*d*_6_. The mass spectra were recorded on a Waters ACQUITY TQD LC-MS/MS mass spectrometer. Crystal structures were determined using a Bruker SMART APEX CCD X-ray single-crystal diffractometer. The MTT assay was performed using an American Thermo FC microplate.

Chemicals and solvents were obtained from commercial sources. Thin-layer chromatography (TLC) was performed on pre-coated silica gel aluminum-backed plates (Merck F254, Darmstadt, Germany). Chromatograms were visualized under UV light at 254 and 365 nm.

### 3.2. MTT Assay

The MTT (3-(4,5-dimethylthiazol-2-yl)-2,5-diphenyl-2*H*-tetrazolium bromide) assay was performed as described by Ho *et al.* [19]. Cells were seeded in a 96-well plate at a concentration of 1.0 × 10^4^ cells/well and allowed to adhere overnight. Five replicates were prepared for each treatment and cultured for 48 or 72 h. After 20 μL of MTT (5 mg/mL) was added to each well, the cells were cultured for another 4 h. The supernatant was discarded. After 150 μL of DMSO was added to each well, the samples were incubated for 30 min and then swirled for 10 min. The absorbance *A* at 570 nm was measured using a microplate reader. Experiments were repeated three times.

### 3.3. Synthesis

#### 3.3.1. Carbonic Acid 3,5-bis-(2,2,2-Trichloroethoxycarbonyloxy)-2-{3-[4-(2,2,2-trichloroethoxy-carbonyloxy) phenyl] propionyl}phenyl Ester 2,2,2-Trichloroethyl Ester (**7**)

2,2,2-Trichloroethoxycarbonyl chloride (10 mL) was slowly added to a solution of phloretin (**1**, 2.74 g, 10 mmol) in acetone (30 mL) in an ice bath and then heated at reflux for 3 h. After phloretin was observed to have disappeared by TLC, the residue obtained by evaporation of the solvent was purified by column chromatography using a binary solvent mixture of petroleum ether-ethyl acetate (5:1) to obtain **7** as a white solid (6.4 g, 66%). MS *m/z*: 993.48 [M+Na]^+^; ^1^H-NMR (CDCl_3_, 300 MHz) δ: 7.27 (t, 4H, Ph), 7.12 (d, 2H, *J* = 5.5 Hz, Ph), 4.86 (s, 8H, CH_2_), 3.18 (t, 2H, CH_2_), 2.99 (t, 2H, CH_2)_. ^13^C-NMR (DMSO-d_6_, 75 MHz): 197.6, 152.0, 151.4, 151.0, 150.9, 148.8, 147.6, 138.7, 129.5, 124.9, 120.8, 120.7, 115.1, 94.5, 94.1, 76.9, 76.6, 76.5, 75.3, 44.9, 28.3.

#### 3.3.2. 5,7-Dihydroxy-3-(4-hydroxybenzyl)-2-trifluoromethylchromen-4-one (**10**)

Trifluoroacetic anhydride (10 mL) was added to a mixture of phloretin (**1**, 2.74 g, 10 mmol) and triethylamine (10 mL) in acetone (50 mL) and heated at reflux for 5 h. After a new compound was observed by TLC, the reaction mixture was diluted with water (50 mL) and extracted with dichloromethane (3 × 50 mL). The organic phases were collected and dried over anhydrous magnesium sulfate. The solution was filtered under vacuum and purified by column chromatography using a binary solvent mixture of petroleum ether-ethyl acetate (5:1-3:1) to afford **10** (2.74 g, 78%). MS *m/z*: 352.98 [M+H]^+^; ^1^H-NMR (DMSO-*d*_6_, 300 MHz) δ: 12.20 (s, 1H, OH), 11.22 (s, 1H, OH), 9.24 (s, 1H, OH), 7.00 (d, 2H, *J* = 2.0 Hz, Ph), 6.68 (d, 2H, *J* = 2.5 Hz, Ph), 6.42 (s, 1H, Ph), 6.29 (s, 1H, Ph), 3.82 (t, 2H, CH_2_). ^13^C-NMR (DMSO-d_6_, 75 MHz): 180.4, 165.5, 161.3, 156.3, 155.8, 147.6, 147.3, 128.8, 127.7, 121.8, 115.1, 103.7, 99.7, 93.9, 26.6.

### 3.4. X-ray Crystallographic Data

CCDC 1026553 and 1026554 contain the supplementary crystallographic data for this paper. These data can be obtained free of charge via http://www.ccdc.cam.ac.uk/conts/retrieving.html (or from the CCDC, 12 Union Road, Cambridge CB2 1EZ, UK; Fax: +44 1223 336033; E-mail: deposit@ccdc.cam.ac.uk).

### 3.5. Characterization of Compounds ***2**–**6***, ***8***, ***9*** and ***11***

*1-(2-Hydroxy-4,6-dimethoxy-phenyl)-3-(4-methoxy-phenyl)-propan-1-one* (**2**): ESI-MS *m/z*: 317.11 [M+H]^+^, 340 [M+Na]^+^; ^1^H-NMR (CDCl_3_, 300 MHz) δ 2.900 (t, 2H, CH_2_), 3.252 (t, 2H, CH_2_), 3.705 (s, 9H, OCH_3_), 5.919 (s, 1H, Ph), 6.070 (s, 1H, Ph), 6.852 (d, 2H, Ph), 7.140 (d, 2H, Ph), 14.028 (s, 1H, OH). ^13^C-NMR (DMSO-*d*_6_,75 MHz): 204.45, 165.79, 165.60, 162.40, 155.39, 131.30, 129.11, 115.06, 105.49, 93.79, 90.87, 56.06, 55.64, 45.67, 29.31.

*1-(2-Hydroxy-4,6-dimethoxy-phenyl)-3-(4-hydroxy-phenyl)-propan-1-one* (**3**): ESI-MS *m/z*: 303.62 [M+H]^+^; ^1^H-NMR (DMSO-*d*_6_, 300 MHz) δ 2.936 (t, 2H, CH_2_), 3.243 (t, 2H, CH_2_), 3.801 (s, 3H, OCH_3_), 3.821 (s, 3H, OCH_3_), 5.338 (s, 1H, OH), 5.919 (s, 1H, Ph), 6.071 (s, 1H, Ph), 6.779 (d, 2H, Ph), 7.100 (d, 2H, Ph), 14.094 (s, 1H, OH).

*1-(2,4-Dihydroxy-6-methoxy-phenyl)-3-(4-hydroxy-phenyl)-propan-1-one* (**4**): ESI-MS *m/z*: 289.04 [M+H]^+^; ^1^H-NMR (DMSO-*d*_6_, 300 MHz) δ 2.778(t, 2H, CH_2_), 3.220 (t, 2H, CH_2_), 3.741 (s, 3H, OCH_3_), 5.956 (s, 2H, Ph), 6.661 (d, 2H, Ph), 7.009 (d, 2H, Ph), 9.141 (s, 1H, OH),12.321 (s, 2H, OH). ^13^C-NMR (DMSO-*d*_6_, 75 MHz): 169.22, 165.43, 163.96, 155.38, 131.48, 129.12, 115.04, 104.55, 93.20, 55.31, 45.58, 29.29.

*Acetic acid 4-[3-oxo-3-(2,4,6-triacetoxy-phenyl)-propyl]-phenyl ester* (**5**): ESI-MS *m/z*: 443.28 [M+H]^+^; ^1^H-NMR (CDCl_3_, 300 MHz) δ 2.120 (s, 6H, CH_3_), 2.270 (s, 6H, CH_3_), 2.974 (t, 2H, CH_2_), 3.070 (t, 2H, CH_2_), 6.917 (s, 2H, Ph), 7.003 (d, 2H, Ph), 7.212 (d, 2H, Ph).

*3-(4-Benzyloxy-phenyl)-1-(2,4-bis-benzyloxy-6-hydroxy-phenyl)-propan-1-one* (**6**): ESI-MS *m/z*: 545.35 [M+H]^+^; ^1^H-NMR (CDCl_3_, 300 MHz) δ 2.846 (t, 2H, CH_2_), 3.242 (t, 2H, CH_2_), 5.036 (s, 6H), 6.112 (m, 2H, Ph), 6.836 (m, 4H, Ph), 7.206–7.428 (m, 15H, Ph), 14.037 (s, 1H, OH). ^13^C-NMR (DMSO-*d*_6_, 75 MHz): 204.32, 165.74, 164.51, 162.77, 162.23, 161.43, 160.84, 156.37, 141.02, 137.32, 133.04, 128.96, 128.55, 128.47, 128.39, 128.26, 128.16, 127.96, 127.85, 127.51, 114.51, 106.70, 105.80, 105.20, 94.84, 92.59, 89.76, 71.03, 69.75, 69.10, 45.48, 28.87.

*Acetic acid 4-[3-acetoxy-3-(2,4,6-triacetoxy-phenyl)-allyl]-phenyl ester* (**8**): ESI-MS *m/z*: 485.5 [M+H]^+^; ^1^H-NMR (CDCl_3_, 300 MHz) δ 2.151 (s, 3H, CH_3_), 2.261 (s, 3H, CH_3_), 2.322 (s, 6H, CH_3_), 2.427 (s, 3H, CH_3_), 3.836 (s, 2H, CH_2_), 5.799 (t, 1H, CH), 6.979 (m, 2H, Ph), 7.166-7.260 (m, 4H, Ph). ^13^C-NMR (CDCl_3_, 75 MHz): 169.22, 153.57, 148.67, 129.35, 128.77, 121.81, 121.59, 113.86, 109.22, 20.86, 20.79, 20.42, 18.18.

*5,7-Dihydroxy-3-(4-hydroxy-benzyl)-2-methyl-chromen-4-one* (**9**): ESI-MS *m/z*: 299.09 [M+H]^+^; ^1^H-NMR (DMSO-*d*_6_, 300 MHz) δ 2.384(s, 3H, CH_3_), 3.670 (s, 2H, CH_2_), 6.172 (s, 1H, Ph), 6.303 (s, 1H, Ph), 6.660 (d, 2H, Ph), 7.030 (d, 2H, Ph), 9.162 (s, 1H, OH), 10.767 (s, 1H, OH), 12.950 (s, 1H, OH).

*Acetic acid 4-(7-acetoxy-5-hydroxy-2-methyl-4-oxo-4H -chromen-3-ylmethyl)-phenyl ester* (**11**): ESI-MS *m/z*: 383 [M+H]^+^; ^1^H-NMR (CDCl_3_, 300 MHz) δ 2.273 (s, 3H, CH_3_), 2.316 (s, 3H, CH_3_), 2.401 (s, 3H, CH_3_), 3.863 (s, 2H, CH_2_), 6.524 (s, 1H, Ph), 6.670 (s, 1H, Ph), 6.976 (d, 2H, Ph), 7.244 (d, 2H, Ph), 12.852 (s, 1H, OH). ^13^C-NMR (DMSO-*d*_6_, 75 MHz): 181.35, 169.02, 168.36, 166.36, 160.70, 156.06, 155.62, 148.78, 136.44, 128.94, 121.66, 118.47, 107.33, 104.91, 101.02, 27.96, 20.89, 20.78, 18.48.

## 4. Conclusions

In conclusion, we synthesized a series of phloretin derivatives and tested their antitumor activities toward the MDA-MB-231, SPC-A1, A549, MCF-7 and EC109 cell lines. Most of the tested compounds exhibited moderately strong cytotoxicity toward the five cell lines. Notably, **7** exhibited greater cytotoxicity compared to docetaxel toward the five cell lines tested. Compounds **3** and **4** also demonstrated excellent anticancer activity. Therefore, these derivatives may hold great promise as therapeutic agents for the treatment of human cancers.

## Supplementary Materials

Supplementary materials can be accessed at: http://www.mdpi.com/1420-3049/19/10/16447/s1.

## Supplementary Files

Supplementary File 1
