# Supplementary material for: Synthesis, Crystal Structure, and Biological Evaluation of a Series of Phloretin Derivatives
Source: Molecules. 2014 Oct 13;19(10):16447–57. doi: 10.3390/molecules191016447 (PMC6271035; doi:10.3390/molecules191016447)
Supplement: Supplementary File 1 [file molecules-19-16447-s001.pdf]

Supplementary Materials

Crystal Data

Acetic acid 4-[3-acetoxy-3-(2,4,6-triacetoxyphenyl)-allyl]-phenyl ester (**8**) ($C_{25}H_{24}O_{10}$): M = 484.44, monoclinic, space group P2(1)/c, a = 11.875(6) Å, b = 16.159(8) Å, c = 13.910(7) Å, α = 90.00(7)°, β = 112.101(7)°, γ = 90.00°, V = 2473(2) A^3, Z = 4, Dc = 1.301 mg/m^3, F(000) = 1016, T = 296(2) K. A crystal with approximate dimensions of 0.20 × 0.15 × 0.10 mm^3 was mounted on a glass fiber in a random orientation. Crystallographic data were collected with a Siemens Smart-CCD diffractometer with graphite-monochromated MoKa radiation (k = 0.71073 Å). A total of 12146 reflections was measured by ω scan technique at 296(2) K within 1.9 ≤ θ ≤ 25.0°, of which 4365 were independent with Rint = 0.0396, and 2363 were observed with I ≥ 2σ (I). The structure was solved by Direct Methods and refined by full-matrix least squares on F^2 with anisotropic displacement parameters for all non-hydrogen atoms using Shelxtl-97 program package. The hydrogen atoms were added in idealized geometrical positions. Final R indices [I ≥ 2σ (1)]: R1 = 0.0821, wR2 = 0.2511.

Acetic acid 4-(7-acetoxy-5-hydroxy-2-methyl-oxo-4H-chromen-3-ylmethyl)-phenyl ester (**11**) ($C_{21}H_{18}O_7$): M = 382.35, monoclinic, space group P2(1)/c, a = 7.8575(11) Å, b = 14.470(2) Å, c = 34.172(5) Å, α = 90.00°, β =101.719(4) °, γ = 90.00°, V = 3804.3(9) A^3, Z = 8, Dc = 1.335 mg/m^3, F(000) = 1600, T = 296(2) K. A crystal with approximate dimensions of 0.33 × 0.24 × 0.15 mm^3 was mounted on a glass fiber in a random orientation. Crystallographic data were collected with a Siemens Smart-CCD diffractometer with graphite-monochromated MoKa radiation (k = 0.71073 Å). A total of 18857 reflections was measured by ω scan technique at 296(2) K within 1.9 ≤ θ ≤ 25.1°, of which 6723 were independent with Rint = 0.0736, and 3332 were observed with I ≥ 2σ (I). The structure was solved by Direct Methods and refined by full-matrix leastsquares on F^2 with anisotropic displacement parameters for all non-hydrogen atoms using Shelxtl-97 program package. The hydrogen atoms were added in idealized geometrical positions. Final R indices [I ≥ 2σ (1)]: R1 = 0.1081, wR2 = 0.2060.
